# Effects and molecular mechanism of inhibiting p53 signaling pathway by NSUN4 on the resistance to BCL-2 inhibitor for diffuse large B-cell lymphoma

**DOI:** 10.1007/s10238-026-02117-3

**Published:** 2026-03-22

**Authors:** Yuanfei Shi, Heli Wen, Tianle Cao, Yanchun Zhao, Yi Xu, Jianai Sun, Xiaolong Zheng, Jie Jin, Hongyan Tong, Wanzhuo Xie

**Affiliations:** 1 Department of Hematology, The First Affiliated Hospital, College of Medicine, Zhejiang University, No. 79 Qingchun Road, Hangzhou, 310003 Zhejiang China; 2 Departments of Gastroenterology, The First Affiliated Hospital of Wenzhou Medical University, Wenzhou, China; 3 Clinical Medicine Department of Xinjiang Second Medical College, Xinjiang, China; 4 Department of Hematology, Peking Union Medical College Hospital, Chinese Academy of Medical Sciences, Peking Union Medical College, Beijing, China

**Keywords:** Diffuse large B-cell lymphoma, NSUN4, Venetoclax resistance, Apatinib, p53 signaling pathway

## Abstract

A significant proportion of patients with recurrent and refractory diffuse large B-cell lymphoma (DLBCL) exhibit high levels of BCL-2 expression. However, some of these patients are resistant to BCL-2 inhibitors, and the underlying mechanisms remain unclear. In venetoclax-resistant DLBCL cell line, a Cell Counting Kit-8 (CCK8) assay was used to determine the half-maximal inhibitory concentration (IC_50_) value. Flow cytometric was performed to determine mitochondrial membrane potential (MMP), reactive oxygen species (ROS), and cell apoptosis rates, respectively. The construction of NSUN4 knockdown/overexpression cell line using CRISPR-Cas9 technology. KEGG pathway enrichment analysis indicated that NSUN4 modulates venetoclax resistance. Furthermore, we used Western blotting to explore the mechanism underlying venetoclax-resistant. Finally, the antileukemic activity was further evaluated in an in vivo xenograft model. We identified that NSUN4, an m5C methyltransferase, is highly expressed not only in the venetoclax-resistant cell line but also in the lymph nodes of recurrent and refractory DLBCL patients primarily by inhibiting the p53 signaling pathway, although the precise mechanism warrants further investigation, and is correlated with poor prognosis. Moreover, we discovered that Apatinib could reduce NSUN4 expression and effectively reverse venetoclax resistance. These findings suggest that NSUN4 is a critical target for overcoming venetoclax resistance in DLBCL patients. Our study reveals the role of NSUN4 and the p53 signaling pathway in venetoclax resistance at the molecular, cellular, and animal levels. Understanding how m5C methylation mediates venetoclax resistance and regulates the p53 pathway will provide a theoretical foundation for overcoming venetoclax resistance in patients with recurrent and refractory DLBCL.

**Supplementary Information:**

The online version contains supplementary material available at 10.1007/s10238-026-02117-3.

## Introduction

Diffuse large B-cell lymphoma (DLBCL) is the most common subtype of non-Hodgkin lymphoma (NHL) in adults, accounting for approximately 30%-40% of NHL cases [1]. According to the latest reports in 2024, the current first-line treatment for DLBCL is the R-CHOP regimen, which combines CD20 monoclonal antibody with cyclophosphamide, doxorubicin, vincristine, and prednisone. This regimen achieves complete remission (CR) in 60% of patients, significantly improving prognosis [2, 3]. However, nearly one-third of DLBCL patients experience drug resistance or early recurrence after following first-line treatment, resulting in a poor prognosis. Research indicates that high genetic heterogeneity are the primary causes of the challenges in treatment and recurrence of DLBCL. According to the traditional WHO classification, DLBCL is categorized into germinal center B cell-like (GCB) and activated B cell-like (ABC) and three types of uncertain cell origins based on Cell of Origin (COO) [1, 4, 5]. However, with the advent of targeted therapies, this classification method is no longer sufficient to meet the individualized treatment needs of DLBCL. In 2021, Professor Laurie H Sehn’s team introduced a novel molecular typing system for DLBCL based on genetic background features (A53, MCD, N1, BN2, ST2, and EZB), aiming to shift the treatment paradigm from R-CHOP dominated first-line therapy to individualized and precise targeted therapy [6]. Therefore, in-depth investigation of the molecular regulatory mechanisms underlying DLBCL development and progression, as well as exploration of new, precise targeted therapy strategies, is crucial for improving patient survival rates and remain a primary research focus.

Currently, inhibitors targeting key molecules involved in the progression of hematological malignancies, such as IDH1/2 inhibitors, BCL-2 inhibitors, and vascular endothelial growth factor receptor 2 (VEGFR2) inhibitors, are increasingly being used and have demonstrated promising results in hematological diseases [7–9]. Among these, Venetoclax (ABT-199), a highly selective inhibitor of BCL-2, plays an important role in mitochondrial apoptosis [10]. Most acute myeloid leukemia (AML) stem cells exhibit abnormally high expression of BCL-2 and depend on BCL-2 for survival [11]. Approximately one-third of DLBCL patients have BCL-2 translocations and high BCL-2 expression, which are associated with poor prognosis [12]. Targeting BCL-2 family proteins has become a key clinical treatment strategy for hematological malignancies, including DLBCL [13], and has been approved for treating patients with chronic lymphocytic leukemia (CLL) and small lymphocytic lymphoma (SLL) [14, 15]. Significant progress has been made in the combining azacitidine, decitabine, or low-dose cytarabine for treating adult AML [16–18]. Recently, a phase I trial of venetoclax monotherapy in relapsed/refractory(R/R) NHL found an overall response rate (ORR) for DLBCL patients is only 18% [19]. The reasons for the lower ORR in DLBCL remain unclear. Research suggests that upregulation of anti-apoptotic proteins MCL-1 and BCL-XL or alterations of mitochondrial metabolic pathways are significant contributing factors [20, 21]. Improving the efficacy of venetoclax and identifying new biomarkers to predict patient response to venetoclax treatment are urgent clinical challenges. In the era of multiple targeted drug combination applications, the efficacy and underlying mechanisms of venetoclax and other targeted drug therapies require further investigation. Therefore, treatment plans based on BCL-2 inhibitors for DLBCL still need continuous exploration.

In recent years, the widespread application of venetoclax in hematological malignancies has garnered significant attention [22]. However, the emergence of venetoclax resistance limits its clinical utility. Our preliminary research indicates that venetoclax resistance may be associated with the high expression of anti-apoptotic proteins, such as MCL-1, BCL-XL, VEGFR2, or the alteration of mitochondrial metabolic pathways [23]. Additionally, our previous studies have demonstrated that the novel tyrosine kinase inhibitor Apatinib can enhance the chemotherapy sensitivity of the BCL-2 selective inhibitor venetoclax in DLBCL [24]. Apatinib is a new generation of small molecule tyrosine kinase inhibitors targeting vascular endothelial growth factor receptor 2 (VEGFR2), has shown therapeutic effects in various tumors and is clinically used for advanced treatment of multiple solid tumors [25]. Studies have reported that Apatinib also exhibits cytotoxic effects on DLBCL cells. Thus, combination drug therapy appears to be a promising approach to overcoming venetoclax resistance [26]. With the efficient and rapid pace of economic and societal development, the use of existing anti-tumor drugs on the market can significantly reduce the time and cost of developing new anti-tumor drugs. Consequently, researchers increasingly accept the combination use of existing anti-tumor drugs and the investigation of their novel anti-tumor mechanisms. We previously established a venetoclax-resistant (venetoclax-R) DLBCL cell line and found that Apatinib effectively reverses venetoclax resistance. RNA-seq analysis revealed that venetoclax resistance is associated with high expression of NSUN4, an important adverse factor affecting patient prognosis. KEGG enrichment analysis indicated that NSUN4 primarily exerts its effect by inhibiting the p53 signaling pathway. The p53 gene regulates processes such as cell cycle, aging, and apoptosis, and its positive expression in DLBCL is an independent prognostic factor [27].

RNA modification is believed to play a significant role in the development and progression of various tumor types. For example, high expression of NSUN4 is associated with methylation and demethylation processes, as well as the survival of liver cancer patients [28]. NSUN4 is a 5-methylcytosine (m5C) methyltransferase responsible for mitochondrial rRNA methylation, and it participates in the assembly of ribosomes and the translation of related molecules in mitochondria by forming stable dimer complexes with MTERF4 [29]. Research has found that NSUN4 is crucial for embryonic development, and its knockout can be lethal to embryos and occasionally inducing myocarditis in mice. Bioinformatics analysis revealed significant differences in NSUN4 distribution and expression between liver cancer tissues and adjacent tissues [30]. However, there is currently limited research on the role of NSUN4 in the occurrence and development of DLBCL by NSUN4. Our preliminary experimental results indicated that NSUN4 was highly expressed in venetoclax-resistant DLBCL cell lines, and its expression in lymph node tissues of DLBCL patients was higher than that in normal tissues. Apatinib effectively reverses venetoclax resistance and reduces NSUN4 expression. Therefore, we speculate that NSUN4 is an important target for inducing venetoclax resistance.

In summary, while it has been established that NSUN4 expression can promote the occurrence and progression of certain tumors, there have been no reports have yet examined its role in DLBCL. Additionally, research on the regulation of NSUN4 expression remains limited. Our preliminary study indicates that the development of venetoclax resistance in DLBCL is associated with high NSUN4 expression. Therefore, we hypothesize that NSUN4 is a key gene involved in venetoclax resistance. This project aims to utilize DLBCL cell lines, primary cell samples, and tumor-bearing mice as research models. Building on existing research, we seek to verify the biological effects of NSUN4 and explore the feasibility of reversing venetoclax resistance both in vitro and in vivo and to elucidate the specific mechanism of NSUN4-mediated venetoclax resistance. The implementation of this project is expected to yield innovative results in targeted therapy for hematological malignancies, especially DLBCL, and holds significant scientific value and application prospects.

## Materials and methods

### Drugs and reagents

The drugs venetoclax and Apatinib were obtained from Selleck Chemicals (Houston, TX, USA). Dimethyl sulfoxide (DMSO) was used as a solvent for preparing the stock solutions of the reagents (Invitrogen, Carlsbad, CA, USA) stored at -20 °C, subsequently diluted to the required concentrations according to the manufacturers’ instructions.

### Cell lines and cell culture

Human DLBCL cell lines OCI-Ly1, OCI-Ly3, OCI-Ly10, OCI-Ly19, and SU-DHL-4 were purchased from ATCC (Rockefeller, MD, USA) and the cell lines were cultured in RPMI-1640 medium (Gibco, Billings, MT, USA). OCI-Ly3 cells were cultured in IMDM (Gibco). Supplemented with 10% fetal bovine serum (HyClone, Thermo Scientific, Waltham, MA, USA) maintained at 37 °C in a humidified CO_2_ incubator. Primary DLBCL samples (*n* = 50) were obtained from the Department of Hematology, the First Affiliated Hospital, College of Medicine, Zhejiang University (Hangzhou, China) between 2023 and 2024. This study was approved by the Ethics Committee of the First Affiliated Hospital of Zhejiang University. All study procedures adhered to the Declaration of Helsinki, and all experiments were conducted with the written informed consent was obtained from all participants.

### Cell viability assay

Cell Counting Kit-8 (CCK-8) assay was used to test the cytotoxic effects of venetoclax and Apatinib on DLBCL cells (Dojindo, Kumamoto, Japan). In brief, cells (2 × 10^4^ cells per well) were seeded in 96-well plates containing a 100 µl of growth medium and treated with different concentrations of venetoclax or Apatinib, either alone or in combination, for 12–24 h. CCK-8 reagent (10 µl per well) were added and incubated for an additional 2–4 h at 37 °C, then the absorbance at 450 nm was measured by a microplate reader (ELx800; BioTek Instruments Inc., Winooski, VT, USA). For each cell line, experiments were conducted in triplicate. Additionally, cells were treated with venetoclax or Apatinib alone or in combination for 24 h in 24-well plates and the results were observed under a light microscope.

### Flow cytometric assay for determining mitochondrial membrane potential (MMP) and reactive oxygen species (ROS)

The apoptosis rate of DLBCL cells was assessed by treating them for 12 and 24 h with different concentrations of venetoclax and Apatinib alone or in combination according to the manufacturer’s instructions. DLBCL cells were harvested and then analyzed using a Novocyte flow cytometer (ACEA Bioscience, San Diego, CA, USA) after staining with Annexin V/PI (Thermofisher, USA) staining for 15 min at room temperature in the dark. Leukocytes were isolated from bone marrow or tissue samples of patients diagnosed with primary DLBCL. We used the Click-iT EdU Kit (Thermofisher) to analyze the cell cycle. Mitochondrial membrane potential (Δψm) loss was detected using the JC-1 Fluorescent Probe Kit (Beyotime Company, Shanghai, China). A ROS Assay Kit (Beyotime, Shanghai, China) was used to measure cellular ROS levels, as previously reported, and the results were expressed as the ratio of the mean fluorescence intensity.

### Clonogenic assay

To verify whether knocking out NSUN4 inhibits colony-forming ability, we conducted a colony-forming assay. DLBCL cells were treated with different concentrations of agents, and then seeded in methylcellulose medium (Methocult H4100, Stem Cell Technologies, Vancouver, BC, Canada) at a density of 500 cells/well for approximately 14 days. Colonies were stained with MTT and their tumor-forming potential was assessed in vivo, and the size of the colonies was observed under a light microscope.

### Western Blot analysis

The OCI-Ly1 cells were lysed on ice in lysis buffer, electrophoresed in 10% SDS-PAGE gel, and then transferred to NC membranes (Millipore, Billerica, MA, USA). A 5% nonfat milk solution in TBS-T was used for blocking the membranes, followed by incubation with primary antibodies (1:1000 in 5% bovine serum albumin in TBS-T) overnight at 4 °C. The membranes were then incubated with secondary HRP-conjugated antibody (1:20000; Multi Sciences Biotech) and visualized using an ECL detection kit (Biological Industries, Beit HaEmek, Israel). The primary antibody against NSUN4 was purchased from Abcam (Cambridge, MA). BCL-XL, BCL-2, MCL-1, and β-actin antibodies were purchased from Cell Signaling Technology (Danvers, MA, USA).

### RNA sequencing

Extract RNA from venetoclax-sensitive cell lines OCI-Ly1-S and OCI-Ly1-R and perform RNA sequencing. RNA sequencing (RNA-seq) was performed through a commercial service (service ID# F20FTSECWLJ3511, BGI, Huada Gene, Wuhan, China). The heatmap was generated using Pheatmap according to the gene expression in different groups. Essentially, differential expression analysis was conducted using DESeq2 [31] with a Q value ≤ 0.05. GO and KEGG enrichment analyses was performed using Phyper based on a Hypergeometric test. The significant levels of terms and pathways were corrected by Q value with a stringent threshold (Q value ≤ 0.05) by Bonferroni [32]. A pathway activity network was constructed using Cytoscape [33] to visualize enriched biological pathways with significance (*P* < 0.05). The computational method used to identify the most important seed genes was Molecular Complex Detection (MCODE). The algorithm we used to screen key genes is called MCODE. It is one of the most widely used algorithms for mining protein complexes. We performed MCODE calculations on the differentially expressed genes, and after clustering, multiple subnetworks were obtained. For each subnetwork, basic information such as the number of nodes, edges, and score can be observed. Among these, the most important subnetwork contains six key genes. Based on the ranking of scores and combined with relevant research background, we determined that the NSUN4 gene is a critical gene associated with venetoclax resistance. This algorithm is more scientific and rigorous compared to conventional observations using fold change (FC) to identify key genes. The genes identified through this algorithm were subsequently validated in molecular experiments, confirming their significance.

### Gene editing and overexpression

CRISPR/Cas9 screen was performed as previous studies. DLBCL cells (OCI-Ly1) were electroporated with ribonucleoprotein (RNP) for genome editing using the Lonza Amaxa Nucleofector II (Lonza, Basel, Switzerland), 4 µM (1:3, Cas9:sgRNA) Alt-R^®^ (Integrated DNA Technologies, Inc) Cas9 RNP complex, and 4 µM Alt-R^®^ Cas9 Electroporation Enhancer (Integrated DNA Technologies, Inc) as described. To form RNPs, chemically synthesized guide RNAs were purchased and prepared according to the manufacturer’s instructions according to the manufacturer’s instructions (Synthego) and purified Cas9 proteins were added (IDT). RNP complexes (5 µl) were combined with 1.5 × 10^5^ DLBCL cells (20 µl) and electroporated following protocol V-001 as per the manufacturer’s instructions. Proteins were harvested six days after electroporation. Genomic DNA was isolated from cell pellets with a Universal Genomic DNA Kit (Cwbio, Beijing, China). Subsequent PCR amplification was carried out using a 2× GoldStar Best Mix kit per the manufacturer’s instructions. The primers used were: sense (5’-AAT GGA CTA TCA TAT GCT TAC CGT TTC-3’) and antisense (5’-AGC AAT TCC ACT CCT TTC AAG TAG − 3’). The resulting PCR amplicons were gel-purified from 2% agarose using a Gel Extraction Kit and then sent for next-generation sequencing on a BGISEQ platform. The generated NGS data were processed with the MAGeCKFlute package, and cumulative frequency was calculated as per established methods.

### Animals

Pre-processed OCI-Ly1 cells were transplanted into 6-8 weeks old female NSG (strain name: NOD. CgPrkdcscid Il2rgtm1Wjl/SzJ) were used for in vivo studies. A randomization method was applied prior to treatment based on tumor caliper measurements. Each animal’s weight was monitored three times a week during treatment at Zhejiang University’s First Affiliated Hospital, Department of Medicine. All experiments were approved by the Ethics Committee for Laboratory Animals of the First Affiliated Hospital, College of Medicine, Zhejiang University (Hangzhou, China) (Reference number: 624) and were conducted following the National Institutes of Health Guide for the Care and Use of Laboratory Animals. OCI-Ly1 xenografts were generated in 20 NSG mice, which were subcutaneously injected into the left flank with 1 × 10^7^ OCI-Ly1 cells per animal, resuspended 1:1 in PBS: Matrigel (Sigma-Aldrich).

Once the tumor volume reached approximately 75 mm^3^, mice were randomly assigned to three treatment groups (*n* = 5 per group): sgCtrl, sgNSUN4, and sgNSUN4 + Vene. Throughout the treatment period, mice were monitored and weighed daily to assess potential toxicity. After two weeks, tumor volume was measured daily using a caliper and calculated with the formula V = (*L×W²*) /2, where L and W represent the tumor length and width, respectively.

### Human Samples

From July 2023 to April 2024, 50 patients with DLBCL and 50 normal controls were included in this study. A detailed diagnosis and treatment history were obtained from their medical records at the Zhejiang Institute of Hematology, Zhejiang University, China. The study was approved by the Ethical Committee of the First Affiliated Hospital of Zhejiang University (Hangzhou, China) approved this study. Patients were informed of the risks and benefits of participating in this study.

### Statistical analysis

All experiments were performed in triplicate when indicated, and the values are expressed as the mean±standard deviation (S.D.). GraphPad Prism 7 software was used for statistical analysis. The Student’s t-test was used to compare the mean between the two groups. Multiple-group comparisons were performed using the one-way analysis of variance (ANOVA) followed by the Bonferroni post hoc test. *P* < 0.05 was considered statistically significant, and different levels were described as *P < 0.05, **P < 0.01, and ***P < 0.001, respectively. All statistical analyses were performed using SPSS version 20.0 software (La Jolla, CA).

## Results

### Establishment and Validation of DLBCL-resistant cell lines

**Fig. 1:**
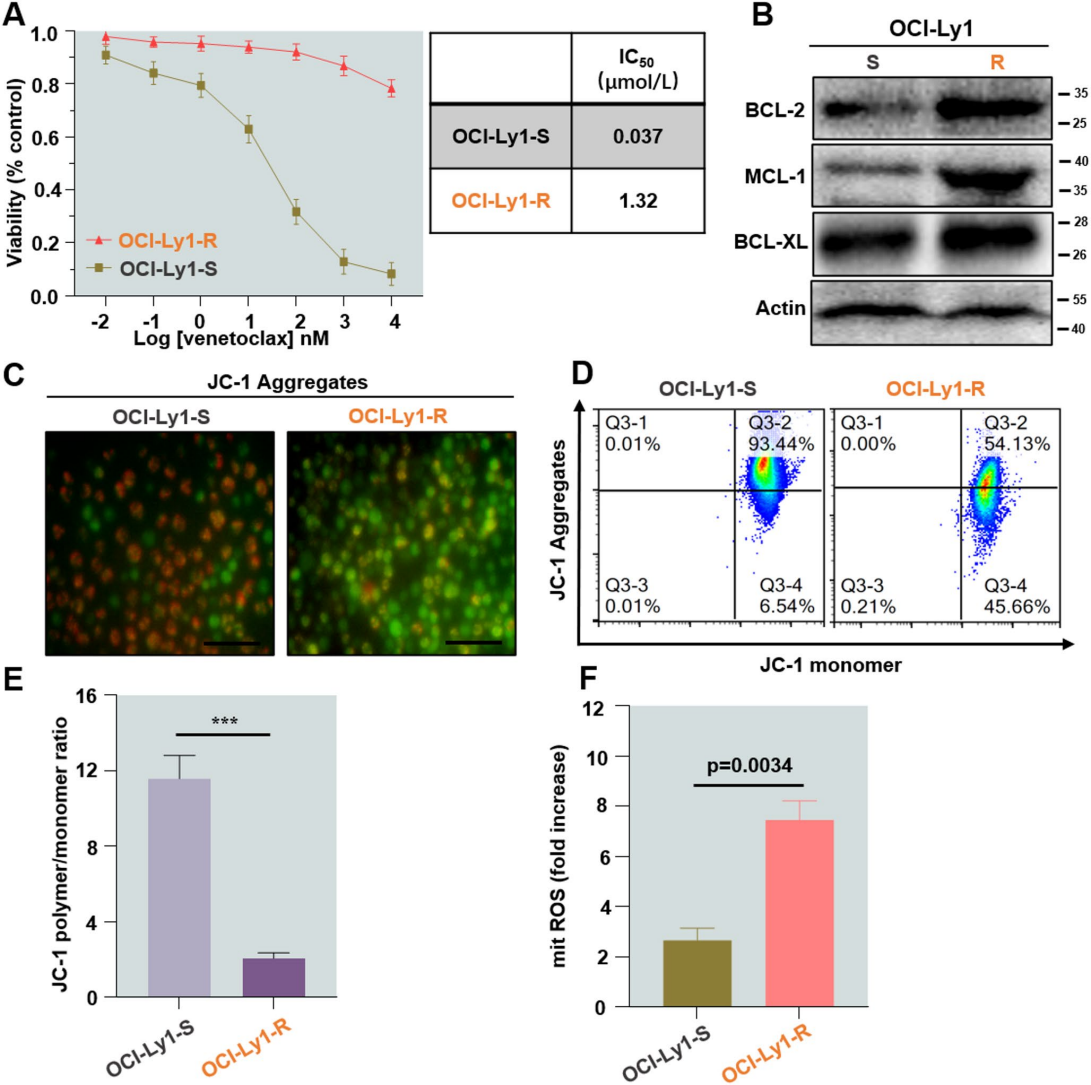
Establishment and validation of DLBCL-resistant cell line OCI-Ly1. **(A)** Cell proliferation curves of OCI-Ly1-S and OCI-Ly1-R cells. **(B)** Western blot analysis of BCL-2, MCL-1, and BCL-XL protein levels in OCI-Ly1-S and OCI-Ly1-R cells. **(C**,** D**,** E)** OCI-Ly1-S and OCI-Ly1-R cells with loss of mitochondrial membrane potential (scale bar: 100 μm). **(F)** Meanwhile, intracellular ROS levels were measured by flow cytometry using the ROS Assay Kit. All experiments were repeated three times (*n* = 3)

First, we established a venetoclax-resistant cell line in OCI-Ly1 and evaluated the inhibitory effect of venetoclax on cell viability using the Cell Counting Kit-8 (CCK-8) assay. The OCI-Ly1-resistance cell line (OCI-Ly1-R) showed a higher half-maximal inhibitory concentration (IC_50_) than the sensitive OCI-Ly1 cells (OCI-Ly1-S). IC_50_ was nearly 35-fold higher in the sensitive cell line compared to the sensitive cell line **(**Fig. 1A**)**. The resistant cells were found to express higher levels of BCL-2, MCL-1, and BCL-XL when compared with their sensitive counterparts **(**Fig. 1B**)**. Additionally, the resistant cells also induced loss of mitochondrial membrane potential (MMP), reflected by markedly decreased fluorescence intensity ratio between JC-1 aggregates and monomers (Fig. 1C-E). To uncover the potential mechanism underlying the generation of resistant cells, flow cytometry was used to detect intracellular Reactive Oxygen Species (ROS) levels. It was found that higher levels of ROS were detected in resistant cells **(**Fig. 1F**)**.

### NSUN4 was significantly enriched in pathway activity networks

**Fig. 2:**
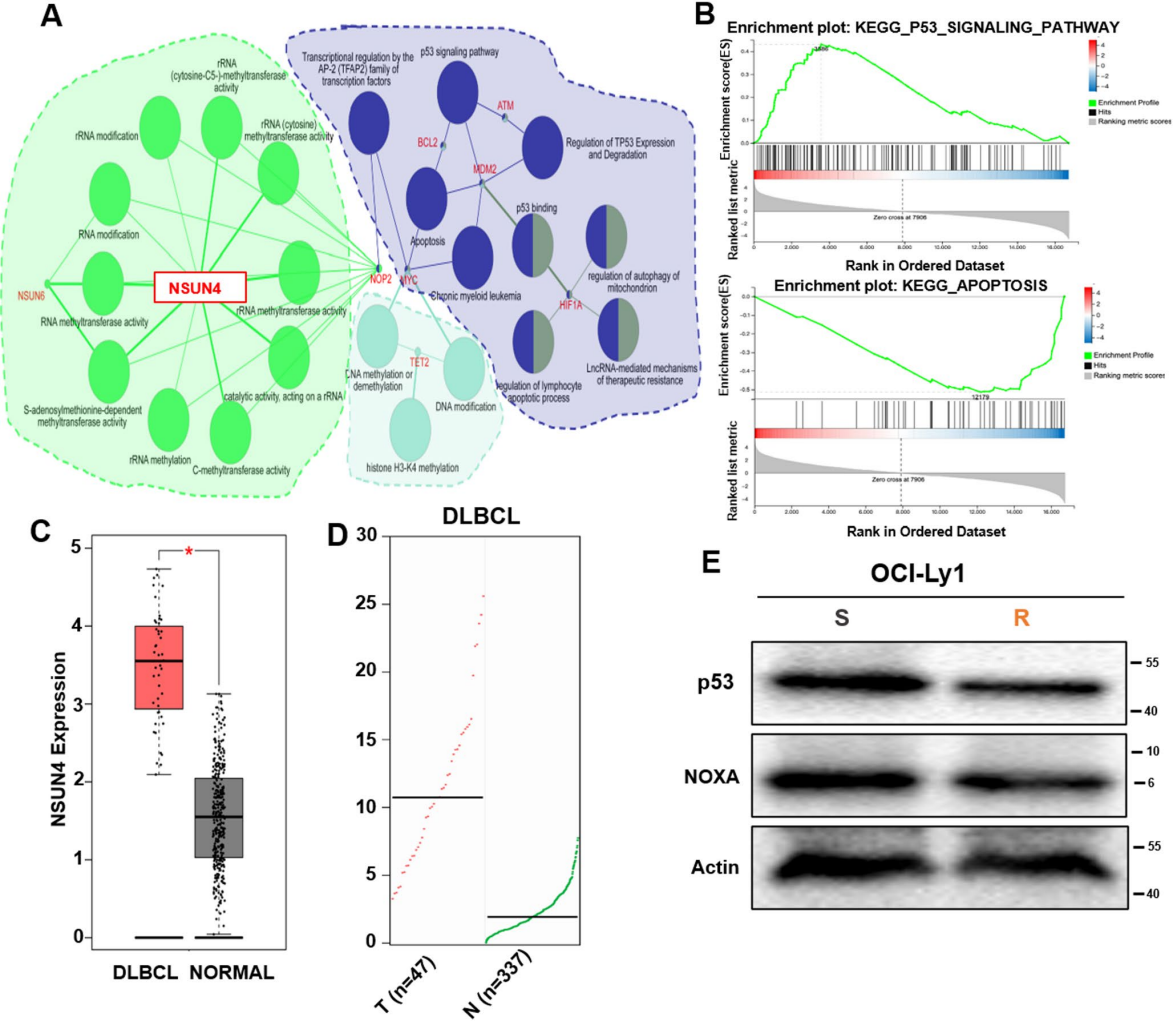
The identification and validation of genes associated with drug resistance. **(A)** Using MCOD to search for resistance genes. **(B)** The enrichment of related signaling pathways. **(C**,** D)** Normal and lymph node tissues express NSUN4 protein levels. **(E)** Western Blot analysis was performed to validate the downregulation efficiency of NSUN4 on p53 in OCI-Ly1-R/S cells. All Western Blot experiments were repeated three times (*n* = 3)

Preliminary research using DLBCL cells allowed us to screen for the molecular target of venetoclax-S/R using RNA-seq. We found that 192 genes were differentially expressed in venetoclax-S/R cells **(S Table 1)**. Six key genes including NSUN4, NOP2, MYC, TET2, MDM2, and HIF1A were significantly enriched according to the pathway activity network, and venetoclax resistance is strongly influenced by NSUN4 **(**Fig. 2A**)**, which primarily promotes cell apoptosis and regulates p53-related signaling pathways **(**Fig. 2B**)**. In hematological malignancies, NSUN4 plays a significant role in tumorigenesis. NSUN4 mRNA expression levels in DLBCL tissues were analyzed at an early stage using the Gene Expression Profiling Interaction Analysis (GEPIA) database revealing that NSUN4 levels were significantly higher in DLBCL patients compared to normal tissues **(**Fig. 2C and D**)**. The RNA-seq result analysis find that venetoclax resistance is strongly influenced by NSUN4, which primarily promotes cell apoptosis and regulates p53-related signaling pathways and its downstream target NOXA (Fig. 2E). Through RNA-seq, we identified NOXA as a downstream target gene regulated by NSUN4. The results from the volcano plot revealed a positive correlation between NSUN4 and NOXA (**S** Fig. 1). Western Blot showed the overexpression of p53 leads to an increase in the expression of its downstream gene, NOXA (**S** Fig. 1). Thus, we hypothesized that venetoclax resistance is related to the high expression of NSUN4 and which acts by inhibiting the p53 signaling pathway.

### NSUN4 was expressed at variable levels in all DLBCL cell lines irrespective of their cell of origin

**Fig. 3:**
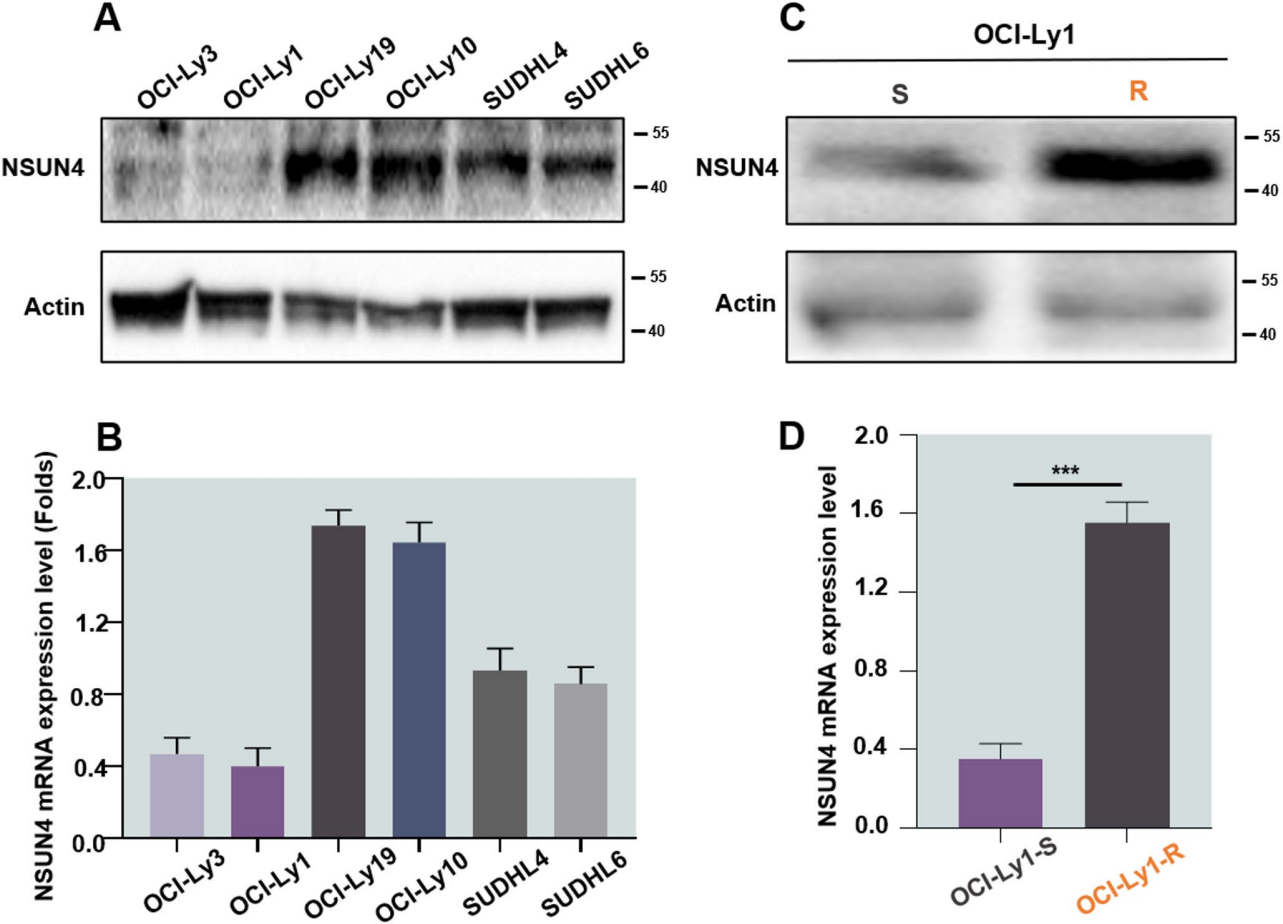
The expression of NSUN4 in DLBCL cell lines. **(A**,** B)** NSUN4 was expressed at variable levels in all DLBCL cell lines irrespective of cell of origin. **(C**,** D)** Validate the expression level of NSUN4 in venetoclax-S/R cell. All experiments were repeated three times (*n* = 3). ***P < 0.001

Using Western blot and RT-qPCR methods, we examined the expression of NSUN4 in various DLBCL cell lines, including OCI-Ly1, OCI-Ly3, OCI-Ly10, OCI-Ly19, as well as SU-DHL4 and SU-DHL6 cells. NSUN4 expression was low in OCI-Ly1 and OCI-Ly3, with the lowest levels observed in OCI-Ly1 **(**Fig. 3A and B**)**. The association between venetoclax resistance and NSUN4 was further confirmed by Western blot and RT-qPCR analyses **(**Fig. 3C and D**)**. Based on these findings, we hypothesized that downregulating NSUN4 could reverse venetoclax resistance.

### NSUN4 gene, an important adverse factor affecting the prognosis of DLBCL patients

**Fig. 4:**
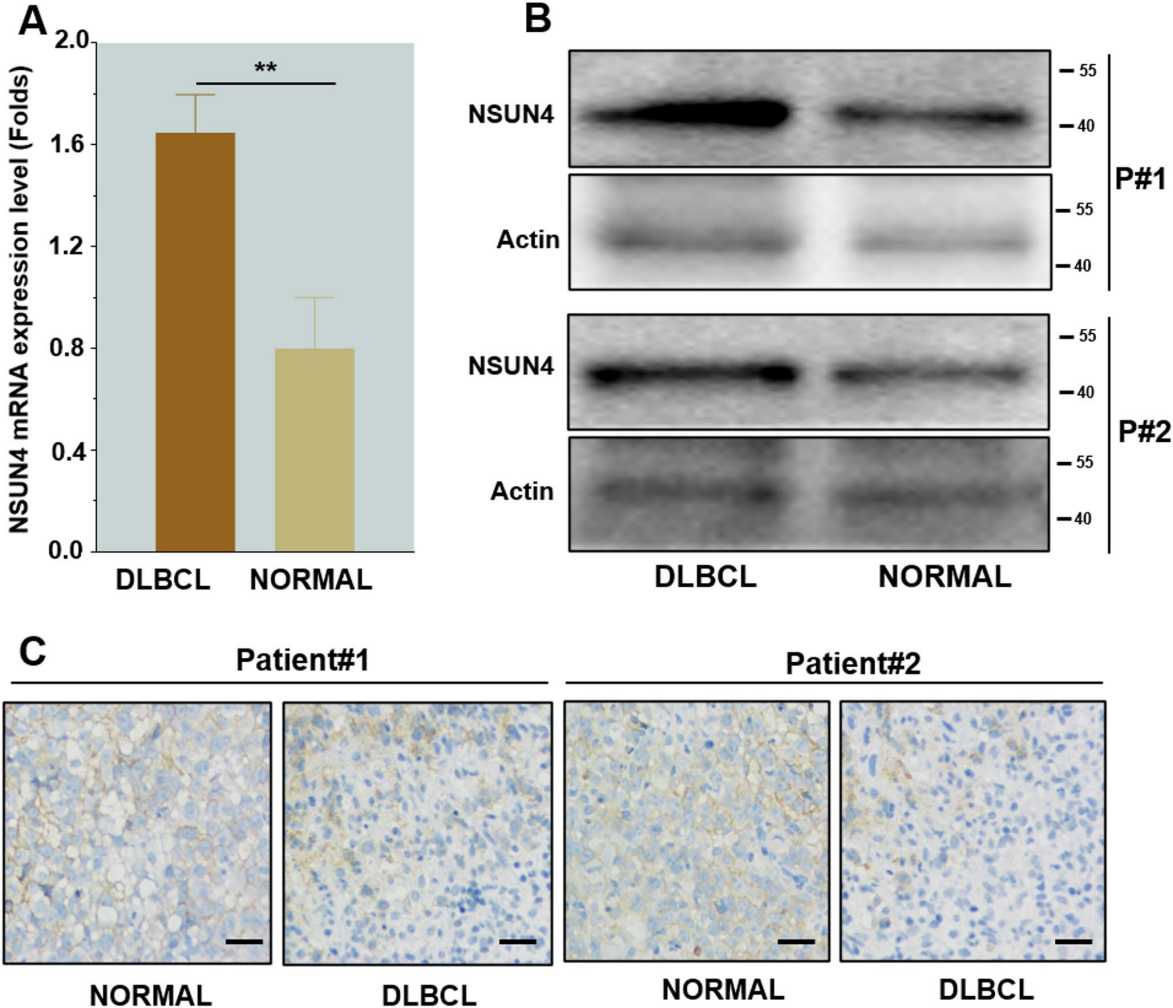
NSUN4 is significantly expressed in DLBCL compared to normal tissues. **(A)** mRNA expression levels of NSUN4 in lymph node tissue and normal tissue. **(B)** Normal and lymph node tissues express NSUN4 protein levels. **(C)** Infiltration of tumor cells in normal tissues and lymph nodes. (scale bar: 20 μm). *P < 0.05, **P < 0.01

Fifty DLBCL patients and fifty patients with lymph node enlargement caused by infection or other tumor metastasis at our center were selected and used as normal tissue controls. RT-qPCR results showed significantly higher NSUN4 expression in DLBCL patients **(**Fig. 4A and B**)**. Additionally, proteins extracted from lymph nodes and normal tissues of two DLBCL patients was analyzed Western blot analysis revealed significant NSUN4 infiltration in the lymph nodes of DLBCL patients compared with normal tissue **(**Fig. 4C**)**. Together, these findings suggest that NSUN4 is an important adverse factor for the prognosis of DLBCL patients.

### Overexpression of NSUN4 predicts poor survival and low responsiveness to venetoclax in DLBCL cell lines

**Fig. 5:**
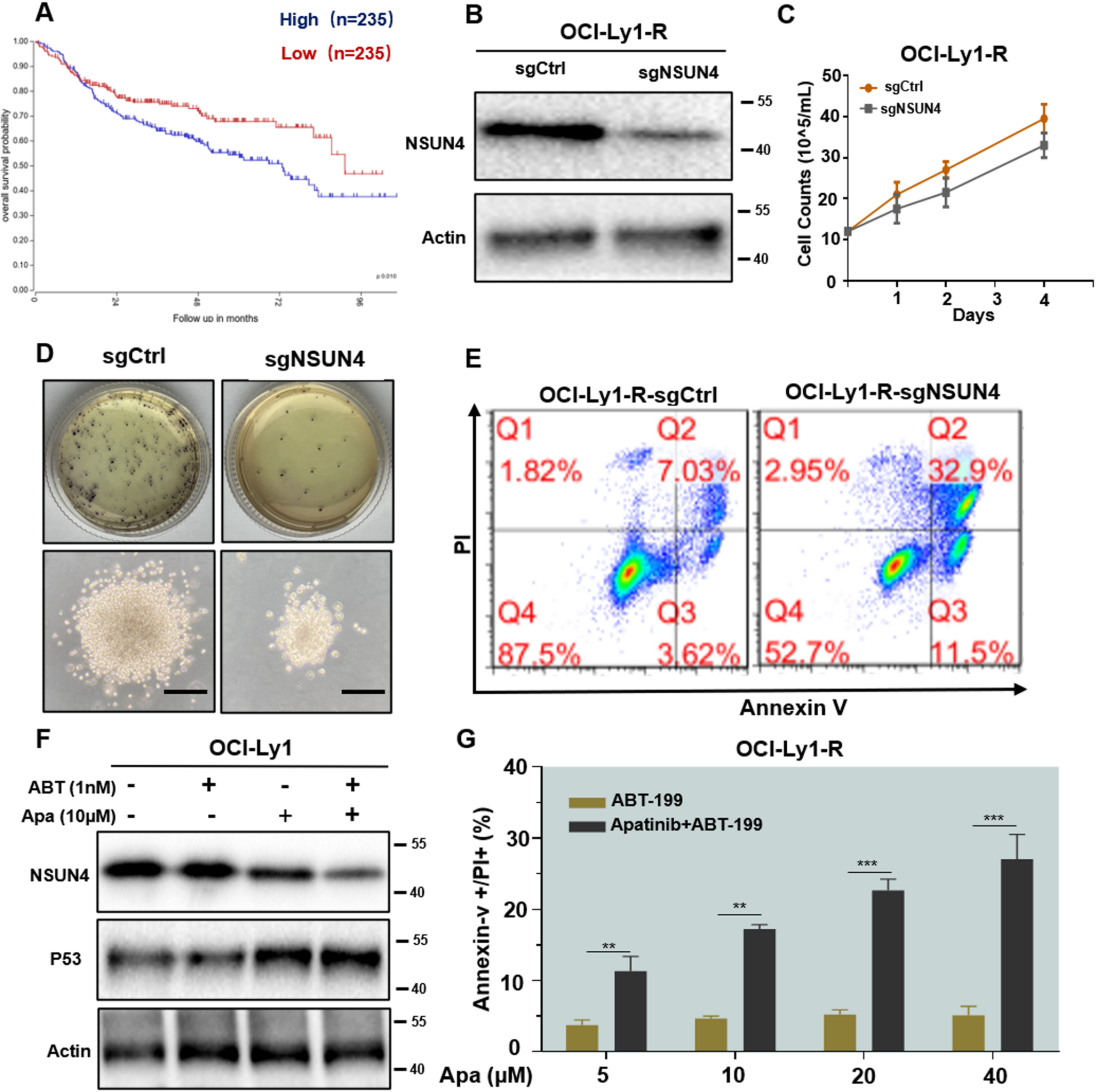
Overexpression of NSUN4 predicts poor survival and low responsiveness to venetoclax in DLBCL cell lines. **(A)** NSUN4 is associated with patient prognosis. **(B)** Protein levels of NSUN4 in OCI-Ly1 cells after CRISPR-Cas9 knockout. **(C)** In a 3-day culture of OCI-Ly1-R cells, proliferation levels were evaluated in the control and sgNSUN4. **(D)** Knocking out NSUN4 affects cell clone formation levels (scale bar: 20 μm). **(E)** NSUN4 knockout leads to a higher level of apoptosis in cells. **(F)** NSUN4 inhibition and activation of the p53 pathway were confirmed by Western blot results for the combination group. **(G)** The combination of Apatinib and venetoclax-R induces apoptosis in the cells. *P < 0.05, **P < 0.01, and ***P < 0.001

NSUN4 was identified as a critical gene of resistance in human DLBCL, and its biological functions were subsequently validated. The GEPIA databases revealed that NSUN4 is a significant adverse factor for prognosis **(**Fig. 5A**)**. Following the knockout of NSUN4 using CRISPR-Cas9 technology, cell proliferation and colony formation experiments showed significant inhibition of DLBCL cell proliferation **(**Fig. 5B and D**)** and an increase in the proportion of cell apoptosis compared to the control group **(**Fig. 5E**)**. Western blot results demonstrated that the combination group could effectively activate the p53 signaling pathway **(**Fig. 5F**)**. In the venetoclax-resistant (R) DLBCL cell line, monotherapy with venetoclax did not induce apoptosis, whereas the combination with Apatinib significantly increased the proportion of apoptosis, with statistical significance **(**Fig. 5G**)**.

### Knocking out NSUN4 impairs tumor-forming capabilities in a xenograft tumor model

**Fig. 6:**
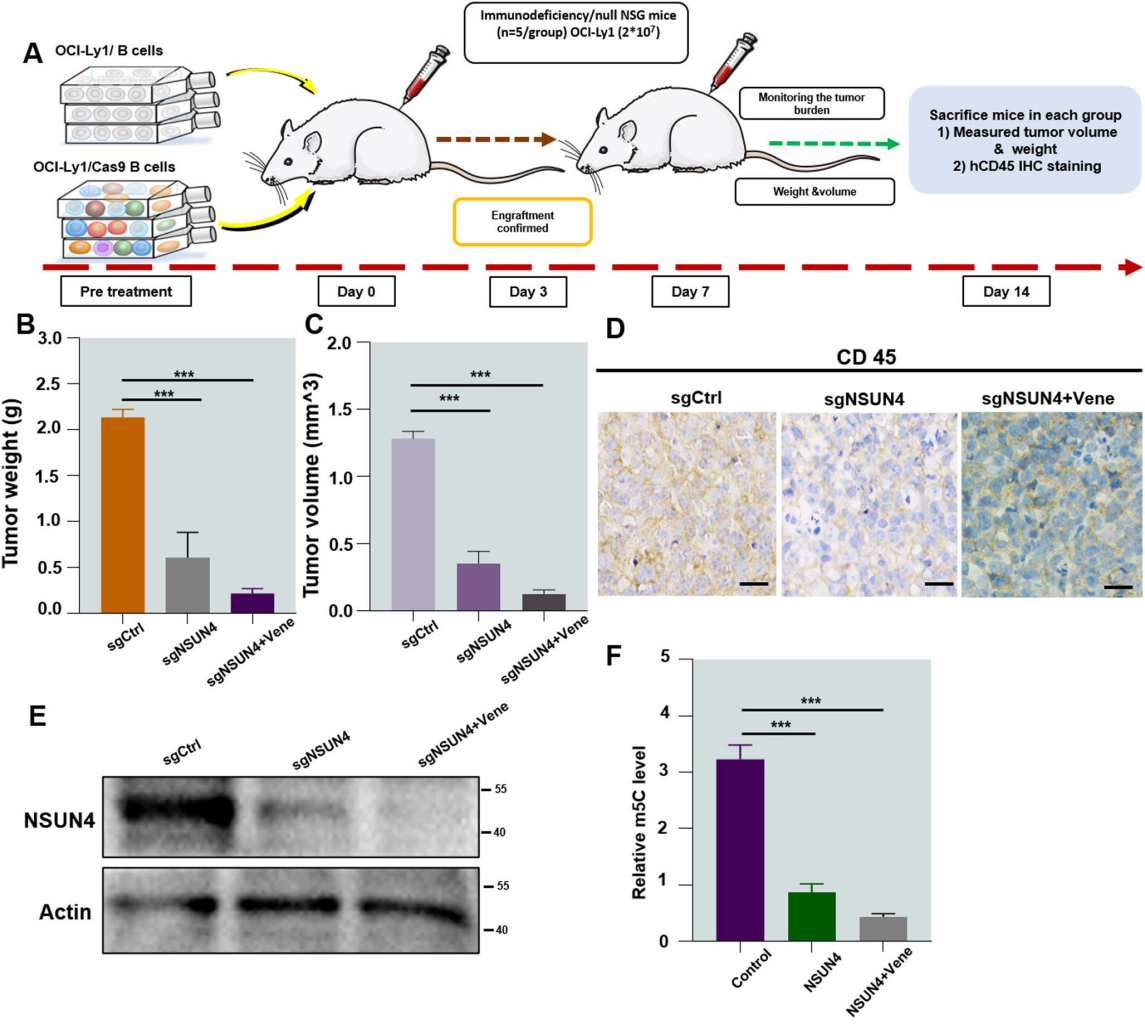
Tumor-forming properties of xenograft models are impaired by knocking out NSUN4 and sgNSUN4 + vene groups. **(A)** Implementation scheme of in vivo experiment. **(B**,** C)** Tumor weight and volume. **(D)** Infiltration of tumor cells was examined by immunohistochemical staining for human CD45 (scale bar: 20 lm). **(E)** Western blot analysis for NSUN4 on tumor homogenate. **(F)** Analyses of mRNA from tumor homogenates detected m5C by dot blot. All western blot experiments were repeated three times (*n* = 3). Data are presented as mean ± SEM. Statistical analyses were performed using an unpaired Student’s t-test. ***P < 0.001

Using a widely adopted xenograft tumor model, we examined the effects of NSUN4 knockout in vivo. Immunodeficient NSG mice were subcutaneously injected with control OCI-Ly1 cells or cells with sgNSUN4. OCI-Ly1 cells with sgNSUN4 or vehicle solution were administered intraperitoneally as schematically depicted **(**Fig. 6A**)**. Compared to the vehicle control, both the sgNSUN4 and sgNSUN4 + vene groups exhibited a significantly lower tumor burden. This reduction was most pronounced in the sgNSUN4 + vene group, as reflected by smaller tumor size along with reduced weight and volume **(**Fig. 6B and C**)**. Histological examination showed that sgNSUN4 and sgNSUN4 + vene treatment inhibited DLBCL cell infiltration compared to the vehicle control **(**Fig. 6D**)**. As expected, a Western blot revealed that NSUN4 expression was inhibited, which is in agreement with the results from in vitro results **(**Fig. 6E**)**. While m5C modification is among the most prominent internal alterations modulating mRNA fate, it is unknown whether this modification participates in the development of venetoclax resistance. Dot blot analysis of total RNA demonstrated a significant decrease in m5C modification in chondrogenic pellets from both the sgNSUN4 and sgNSUN4 + vene groups, as assessed by reduced signal intensity **(**Fig. 6F**)**. These findings suggest that NSUN4 is crucial for venetoclax resistance, as its knockout significantly reduces tumor burden in experimental mice, with m5C methylation modification playing a role in this process.

**Fig. 7:**
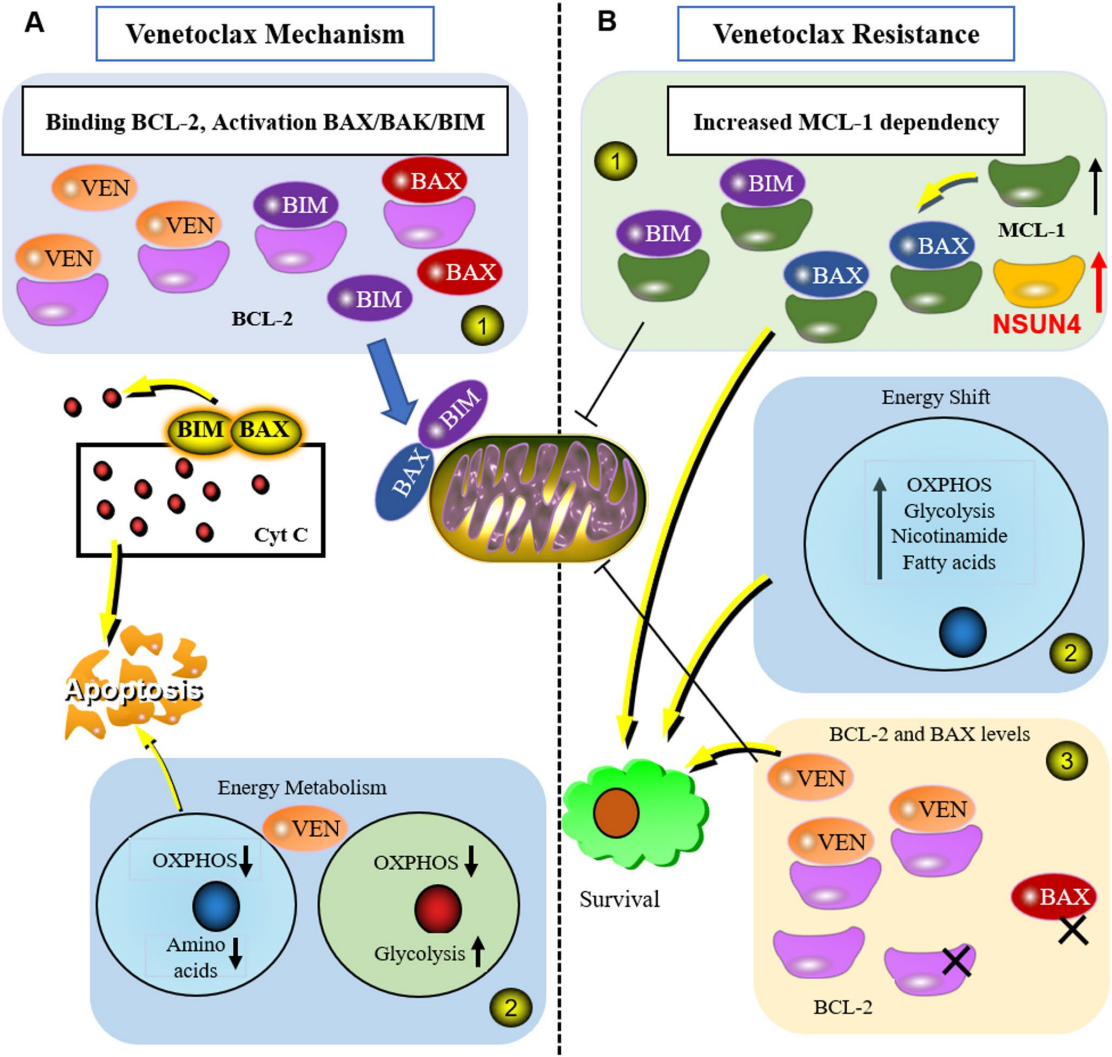
Molecular Mechanisms and Drug Resistance Patterns of venetoclax. **(A)** Mechanism: ① Venetoclax inhibits the interaction between BCL-2 and pro-apoptotic proteins BAX and BIM. BAX and BIM increase mitochondrial outer membrane permeability, release Cyt C, and initiate cell apoptosis. ② Venetoclax reduces OXPHOS and amino acid uptake in leukemia stem cells, while normal HSCs compensate by increasing glycolysis. **(B)** Venetoclax resistance mechanism: ① Increasing dependence on MCL-1 and NSUN4 to prevent mitochondrial localization of BIM and BAX. ② The metabolism of energy is not limited to OXPHOS alone. ③ Venetoclax binding is prevented by the reduction or mutation of BCL-2, while mitochondrial localization is hindered by the mutation of BAX

In summary, NSUN4 expression promotes the occurrence and development of certain tumors, but its role in DLBCL has not been reported. Additionally, research on the regulation of NSUN4 expression is limited. Our preliminary data suggest that venetoclax resistance in DLBCL is associated with high NSUN4 expression. Therefore, it is plausible that NSUN4 plays a significant role in the development of venetoclax resistance. This project utilizes primary cell samples, DLBCL cell lines, and tumor-bearing mice as research materials. Building on existing research, we aim to verify the biological effects of NSUN4 and the feasibility of reversing venetoclax resistance both in vitro and in vivo, and the specific mechanism of NSUN4-mediated venetoclax resistance. The implementation of this project is expected to achieve innovative results in targeted therapy for hematological malignancies, especially DLBCL, with significant scientific and application potential **(**Fig. 7A and B**)**.

## Discussion

Venetoclax is approved for the treatment of acute myelogenous leukemia (AML) and chronic lymphoma leukemia (CLL) with a 1p deletion, and it is also being evaluated for the treatment of multiple myeloma and mantle cell lymphoma subtypes. However, clinical evidence for its efficacy in DLBCL, the most common form of B-cell lymphoma, is limited. Most studies have shown that venetoclax is ineffective as a single agent for DLBCL [34, 35]. Resistance to venetoclax has been demonstrated in clinical trials in various types of cancer, including AML, CLL, multiple myeloma (MM), and mantle cell lymphoma [36, 37]. DLBCL, however, data on venetoclax resistance in DLBCL is lacking. The variety of resistance mechanisms observed in DLBCL underscores the genetic heterogeneity and clinical complexity of this cancer. Patients with DLBCL and their cells often overexpress multiple anti-apoptotic members of the BCL2 family [38–40]. We propose an innovative treatment approach for DLBCL that targets NSUN4 in combination with venetoclax to address venetoclax resistance. Inhibiting NSUN4 and the p53 signaling pathway may help circumvent venetoclax resistance in DLBCL. Our data identifies several vulnerabilities and clinically feasible therapy combinations for venetoclax-resistant DLBCL.

Clonal evolution and subsequent therapeutic resistance in cancer cells are driven by clonal diversity within the cell population [20]. Therapeutic inhibitors often become resistant due to discrete mutations in their target genes [20, 41]. For instance, a BCL2 mutation that impairs the venetoclax binding site in a small cohort of venetoclax-treated CLL patients relapsing on the drug has been observed [42]. However, the number of patients with this mutation was limited, and it occurred only at sub-clonal levels, suggesting that venetoclax resistance is not due to mutational mechanisms alone. Neither our study nor previous research detected this mutation [43].

Systematic characterization of venetoclax-resistance has revealed dramatic alterations in cell line expression profiles and complex changes in genomic sequences associated with venetoclax-resistance [20, 44]. It has been shown that BCL-2 inhibitors consistently resist inhibition due to alterations that overexpress MCL-1 and isolate BIM from BCL-2 inhibitors [45, 46]. Our results, along with functional characterization of resistant cell lines have uncovered that NSUN4 plays an important role in venetoclax resistance and regulates the p53 signaling pathway, with m5C methylation modification also involved in this process. These insights may lead to therapeutic opportunities in B-cell malignancies since mutations affecting these factors have been found across all B-cell malignancies. Based on our findings, BCL-2 inhibitors could be developed for diseases lacking these mutations. Utilizing emerging inhibitors against NSUN4 may be a rational and efficient strategy for combatting venetoclax-resistance.

Dysregulated aberrant RNA epigenetic modification may act as an oncogene or tumor suppressor that contributes to carcinogenesis and tumor growth [47–49]. Epigenetic regulators are promising diagnostic, prognostic, and predictive biomarkers. Studies have suggested m5C methylation genes to be potential biomarkers in DLBCL diagnosis and prognosis [28, 50]. This study focused on RNA epigenetic modifications by examining the expression profiles of m5C RNA methylation regulators to elucidate their significance in DLBCL. Our research indicates that the occurrence of DLBCL is associated with m5C methylation modification.

Our work may open new treatment avenues for patients with lymphoid malignancies resistant to venetoclax and therapeutic vulnerabilities. For instance, our findings enable us to categorize resistance mechanisms in DLBCL patients, allowing for tailored therapeutic approaches accordingly.

## Conclusions

Our study demonstrates that resistance to venetoclax in DLBCL arises from multiple, complex mechanisms. We also identified that venetoclax-resistant DLBCL may be particularly vulnerable to NSUN4 inhibition, which could open up new therapeutic avenues for this challenging disease. Furthermore, combining venetoclax with inhibitors targeting other BCL-2 family members, such as BCLW and BFL1-both of which currently lack specific targeted therapies could serve as an effective strategy to reduce the apoptosis threshold and enhance treatment efficacy. Future studies should explore the relationship between DLBCL molecular subtypes and venetoclax resistance or sensitivity, particularly focusing on mutations linked to pre-existing genetic abnormalities. These mutations could impact the response to different inhibitors or combination therapies, providing insights for more personalized treatment strategies in the future.

## Supplementary Information

Below is the link to the electronic supplementary material.

Supplementary Material 1

Supplementary Material 2: S Fig. 1 Identification and validation of drug resistance-associated genes. (**A**) Volcano plot for identifying the most differentially expressed genes. (**B**) NSUN4 was overexpressing in OCI-Ly1 cells (right).

## Data Availability

The datasets used and/or analyzed during the current study are available from the corresponding author on reasonable request.
