# Supplementary material for: Effects and molecular mechanism of inhibiting p53 signaling pathway by NSUN4 on the resistance to BCL-2 inhibitor for diffuse large B-cell lymphoma
Source: Clin Exp Med. 2026 Mar 22;26(1):204. doi: 10.1007/s10238-026-02117-3 (PMC13038678; doi:10.1007/s10238-026-02117-3)
Supplement: Supplementary file 1 — Supplementary Material 1 [file 10238_2026_2117_MOESM1_ESM.docx]

**S Table 1** 192 genes were differentially expressed in venetocla S/R samples.

| Gene ID | Gene Symbol | Type | log2 (venetoclax-S/R) | Qvalue (venetoclax-S/R) |
| --- | --- | --- | --- | --- |
| 80816 | ASXL3 | mRNA | -1.719916932 | 3.84E-15 |
| 140711 | TLDC2 | mRNA | -4.818685409 | 0.004281059 |
| 23305 | ACSL6 | mRNA | 2.421952747 | 0.001490321 |
| 1608 | DGKG | mRNA | 1.498733655 | 0.032431873 |
| 6696 | SPP1 | mRNA | 3.537221572 | 6.05E-07 |
| 2326 | FMO1 | mRNA | -1.981061851 | 0.001155472 |
| 9900 | SV2A | mRNA | -1.553977395 | 0.026007861 |
| 9452 | ITM2A | mRNA | 1.804966782 | 2.14E-09 |
| 51313 | FAM198B | mRNA | -2.435023097 | 1.25E-27 |
| 219285 | SAMD9L | mRNA | 1.157235084 | 7.19E-07 |
| 55026 | TMEM255A | mRNA | 1.405685635 | 3.84E-04 |
| 89790 | SIGLEC10 | mRNA | 1.487469425 | 7.70E-18 |
| 51186 | TCEAL9 | mRNA | -1.092868707 | 0.03341794 |
| 2331 | FMOD | mRNA | -1.524793149 | 5.72E-122 |
| 7436 | VLDLR | mRNA | 1.272325577 | 1.29E-39 |
| 202151 | RANBP3L | mRNA | -1.324878134 | 1.40E-10 |
| 962 | CD48 | mRNA | 1.323468494 | 1.44E-04 |
| 11185 | INMT | mRNA | 3.263549228 | 5.47E-08 |
| 285220 | EPHA6 | mRNA | 1.282242802 | 0.011734032 |
| 8424 | BBOX1 | mRNA | -1.300787505 | 7.83E-27 |
| 256380 | SCML4 | mRNA | -2.026162773 | 1.33E-18 |
| 3008 | HIST1H1E | mRNA | 3.275216961 | 0.001134261 |
| 22854 | NTNG1 | mRNA | 1.769110213 | 0.001151397 |
| 374860 | ANKRD30B | mRNA | -1.214188635 | 0.010186381 |
| 10157 | AASS | mRNA | 2.18472646 | 0.008526578 |
| 27445 | PCLO | mRNA | -2.104818409 | 4.08E-06 |
| 4101 | MAGEA2 | mRNA | 2.043151393 | 0.012355673 |
| 9922 | IQSEC1 | mRNA | -1.00396371 | 5.91E-04 |
| 4102 | MAGEA3 | mRNA | 1.811887763 | 0.002760557 |
| 4063 | LY9 | mRNA | 1.069029088 | 7.58E-04 |
| 23086 | EXPH5 | mRNA | 1.333871476 | 0.024712204 |
| 29091 | STXBP6 | mRNA | -1.133717194 | 0.046503562 |
| 440279 | UNC13C | mRNA | -4.159923535 | 0.029881759 |
| 114880 | OSBPL6 | mRNA | 1.219884088 | 0.005840742 |
| 115330 | GPR146 | mRNA | -2.336871338 | 0.04361836 |
| 55211 | DPPA4 | mRNA | -1.440238869 | 1.48E-19 |
| 3821 | KLRC1 | mRNA | -1.290608081 | 1.26E-05 |
| 8728 | ADAM19 | mRNA | 1.359336627 | 4.55E-05 |
| 3082 | HGF | mRNA | -1.915606693 | 4.48E-48 |
| 26010 | SPATS2L | mRNA | 1.335976493 | 0.002692831 |
| 10516 | FBLN5 | mRNA | -1.01653016 | 1.23E-16 |
| 64218 | SEMA4A | mRNA | 1.114251521 | 1.42E-08 |
| 375611 | SLC26A5 | mRNA | 2.154212396 | 6.39E-04 |
| 3339 | HSPG2 | mRNA | 4.426144103 | 0.042802269 |
| 10633 | RASL10A | mRNA | -1.015066456 | 0.044980649 |
| 283455 | KSR2 | mRNA | -1.198742878 | 3.21E-59 |
| 401124 | DTHD1 | mRNA | -1.205011642 | 0.008744847 |
| 3162 | HMOX1 | mRNA | 1.354893744 | 1.85E-68 |
| 1487 | CTBP1 | mRNA | 1.671105459 | 3.74E-74 |
| 54453 | RIN2 | mRNA | -1.411086263 | 1.53E-04 |
| 374407 | DNAJB13 | mRNA | -3.479824859 | 0.049275484 |
| 221935 | SDK1 | mRNA | -1.273449782 | 5.42E-15 |
| 285600 | KIAA0825 | mRNA | 1.584823482 | 0.00673433 |
| 83259 | PCDH11Y | mRNA | 2.582504515 | 1.53E-05 |
| 285676 | ZNF454 | mRNA | 1.22655314 | 0.00938863 |
| 81704 | DOCK8 | mRNA | 1.153287773 | 3.02E-11 |
| 51761 | ATP8A2 | mRNA | 4.280667808 | 0.030183003 |
| 4035 | LRP1 | mRNA | -1.271690842 | 4.88E-11 |
| 94241 | TP53INP1 | mRNA | -1.008827577 | 7.89E-102 |
| 9473 | THEMIS2 | mRNA | -1.23020565 | 2.08E-25 |
| 4137 | MAPT | mRNA | 1.175517782 | 0.020165066 |
| 6448 | SGSH | mRNA | -1.158879216 | 0.04292261 |
| 197358 | NLRC3 | mRNA | 1.108870381 | 0.003609374 |
| 107984745 | LOC107984745 | mRNA | -1.03780908 | 1.76E-12 |
| 1534 | CYB561 | mRNA | 1.715729858 | 0.00744316 |
| 9467 | SH3BP5 | mRNA | 1.22649536 | 5.36E-06 |
| 4105 | MAGEA6 | mRNA | 1.169185708 | 0.003856434 |
| 9369 | NRXN3 | mRNA | 3.090934474 | 0.004192947 |
| 5651 | TMPRSS15 | mRNA | -1.121735122 | 0.025911005 |
| 79933 | SYNPO2L | mRNA | 2.157096809 | 0.04256059 |
| 4130 | MAP1A | mRNA | 1.889003416 | 1.69E-162 |
| 3487 | IGFBP4 | mRNA | -1.839003692 | 4.40E-34 |
| 112268437 | LOC112268437 | mRNA | 1.122375959 | 0.018253461 |
| 54766 | BTG4 | mRNA | -1.240290555 | 5.91E-04 |
| 64092 | SAMSN1 | mRNA | 1.422895336 | 7.55E-05 |
| 83871 | RAB34 | mRNA | -1.40349956 | 0.009734769 |
| 11343 | MGLL | mRNA | 1.203735336 | 0.002088529 |
| 115361 | GBP4 | mRNA | 1.921504433 | 3.69E-04 |
| 3217 | HOXB7 | mRNA | -1.329592192 | 0.015307227 |
| 7779 | SLC30A1 | mRNA | 1.28324448 | 4.97E-04 |
| 55530 | SVOP | mRNA | 2.044587855 | 0.019429927 |
| 1284 | COL4A2 | mRNA | 4.111264364 | 0.006065011 |
| 222256 | CDHR3 | mRNA | -1.288609871 | 2.11E-08 |
| 100996747 | LOC100996747 | mRNA | 1.06869199 | 2.19E-07 |
| 93233 | CCDC114 | mRNA | -1.372018828 | 0.004276123 |
| 4685 | NCAM2 | mRNA | 1.821391609 | 0.003729898 |
| 3005 | H1F0 | mRNA | -1.559571305 | 9.50E-09 |
| 8789 | FBP2 | mRNA | -1.40177728 | 1.05E-09 |
| 196415 | C12orf77 | mRNA | -1.428100999 | 1.52E-10 |
| 3043 | HBB | mRNA | 5.369962707 | 0.002493905 |
| 353322 | ANKRD37 | mRNA | -1.326627879 | 0.042983047 |
| 5865 | RAB3B | mRNA | 2.306637493 | 3.08E-07 |
| 143888 | KDELC2 | mRNA | 1.790092805 | 0.043858605 |
| 105373780 | LOC105373780 | mRNA | -1.561443572 | 0.013555147 |
| 915 | CD3D | mRNA | -1.223263448 | 1.13E-04 |
| 6352 | CCL5 | mRNA | 2.539273969 | 0.001207235 |
| 54621 | VSIG10 | mRNA | -1.117878155 | 0.042839829 |
| 5549 | PRELP | mRNA | -1.743649026 | 3.69E-48 |
| 51286 | CEND1 | mRNA | 1.588055107 | 1.51E-17 |
| 729220 | FLJ45513 | lncRNA | -1.920841477 | 0.026603905 |
| 8973 | CHRNA6 | mRNA | 1.842144536 | 5.67E-08 |
| 6510 | SLC1A5 | mRNA | 1.022004217 | 5.08E-106 |
| 100526842 | RPL17-C18orf32 | mRNA | -1.023505024 | 0.006883172 |
| 255394 | TCP11L2 | mRNA | -1.088852688 | 2.14E-08 |
| 100529097 | RPL36A-HNRNPH2 | mRNA | -1.27849104 | 6.14E-12 |
| 57624 | NYAP2 | mRNA | 1.955376787 | 0.021331224 |
| 107986352 | LOC107986352 | mRNA | 1.023200911 | 0.040371116 |
| 735 | C9 | mRNA | -1.044898621 | 1.87E-07 |
| 204801 | NLRP11 | mRNA | 1.228589971 | 2.39E-10 |
| 5896 | RAG1 | mRNA | -1.287955155 | 7.82E-65 |
| 83716 | CRISPLD2 | mRNA | -1.115404444 | 8.80E-09 |
| 8214 | DGCR6 | mRNA | 1.87478845 | 0.022758701 |
| 3039 | HBA1 | mRNA | 5.307081875 | 0.003767618 |
| 54546 | RNF186 | mRNA | -1.851581782 | 2.01E-05 |
| 5996 | RGS1 | mRNA | -1.137671579 | 0.001006659 |
| 57834 | CYP4F11 | mRNA | 3.890658292 | 0.013471168 |
| 84951 | TNS4 | mRNA | -1.391519295 | 4.74E-14 |
| 8715 | NOL4 | mRNA | -1.225400778 | 1.25E-22 |
| 2570 | GABRR2 | mRNA | -1.015976 | 0.011397623 |
| 105372315 | LOC105372315 | other | -1.993643445 | 0.003168539 |
| 800 | CALD1 | mRNA | 4.340616812 | 0.018285559 |
| 8793 | TNFRSF10D | mRNA | -1.274965818 | 7.80E-22 |
| 8681 | JMJD7-PLA2G4B | mRNA | 2.062780113 | 0.016366929 |
| 133 | ADM | mRNA | -1.441621172 | 3.98E-09 |
| 1906 | EDN1 | mRNA | -1.315615713 | 1.51E-05 |
| 8170 | SLC14A2 | mRNA | 1.901208954 | 0.007663288 |
| 3040 | HBA2 | mRNA | 6.275482398 | 8.68E-05 |
| 55076 | TMEM45A | mRNA | -5.685085233 | 7.58E-04 |
| 387758 | FIBIN | mRNA | -1.929547049 | 1.42E-07 |
| 597 | BCL2A1 | mRNA | -1.210062699 | 0.001265094 |
| 7088 | TLE1 | mRNA | -1.243064062 | 3.07E-06 |
| 26108 | PYGO1 | mRNA | -1.021832857 | 6.16E-09 |
| 100533105 | C8orf44-SGK3 | mRNA | -1.215628095 | 1.68E-14 |
| 80059 | LRRTM4 | mRNA | -1.196584551 | 9.95E-21 |
| 345275 | HSD17B13 | mRNA | 2.009623983 | 0.007501279 |
| 54756 | IL17RD | mRNA | 1.386592952 | 0.00312378 |
| 448831 | FRG2 | mRNA | -1.678225533 | 3.21E-59 |
| 8832 | CD84 | mRNA | 1.405057617 | 2.66E-04 |
| 2334 | AFF2 | mRNA | 1.556989197 | 2.12E-28 |
| 10398 | MYL9 | mRNA | 2.755749716 | 0.021980641 |
| 10079 | ATP9A | mRNA | 3.794970738 | 0.019064922 |
| 8553 | BHLHE40 | mRNA | -1.074753461 | 1.44E-09 |
| 4069 | LYZ | mRNA | 1.714728474 | 0.03396861 |
| 8743 | TNFSF10 | mRNA | 2.845486237 | 0.028710756 |
| 2162 | F13A1 | mRNA | -1.403388865 | 5.53E-14 |
| 23024 | PDZRN3 | mRNA | 1.293787443 | 3.15E-07 |
| 162514 | TRPV3 | mRNA | -1.019228167 | 7.06E-15 |
| 388759 | C1orf229 | mRNA | 2.724854347 | 0.002687918 |
| 4629 | MYH11 | mRNA | 1.601039157 | 1.77E-04 |
| 5066 | PAM | mRNA | -1.151874953 | 5.49E-16 |
| 316 | AOX1 | mRNA | 1.230793409 | 1.46E-06 |
| 9447 | AIM2 | mRNA | 1.223857028 | 3.26E-09 |
| 10765 | KDM5B | mRNA | -1.460136085 | 6.50E-13 |
| 79413 | ZBED2 | mRNA | -1.076939365 | 0.002598895 |
| 81491 | GPR63 | mRNA | 1.068315817 | 0.019381156 |
| 9723 | SEMA3E | mRNA | 1.570960364 | 0.008000775 |
| 154141 | MBOAT1 | mRNA | 2.141552613 | 3.23E-07 |
| 100288801 | FRG2C | mRNA | -1.983933614 | 2.80E-04 |
| 5140 | PDE3B | mRNA | -1.034106743 | 4.00E-06 |
| 2549 | GAB1 | mRNA | -1.138065026 | 1.31E-86 |
| 23678 | SGK3 | mRNA | 1.053907652 | 7.20E-10 |
| 171484 | FAM9C | mRNA | -2.27101227 | 7.95E-11 |
| 1612 | DAPK1 | mRNA | -1.461810342 | 2.85E-17 |
| 7431 | VIM | mRNA | 3.345992873 | 0.00497539 |
| 7704 | ZBTB16 | mRNA | -1.627807447 | 9.84E-09 |
| 79887 | PLBD1 | mRNA | 1.104641649 | 0.002308477 |
| 55512 | SMPD3 | mRNA | -1.78206059 | 0.035688191 |
| 1236 | CCR7 | mRNA | -1.913967899 | 1.08E-05 |
| 158471 | PRUNE2 | mRNA | -1.105641156 | 1.22E-05 |
| 29948 | OSGIN1 | mRNA | 3.39514898 | 1.32E-12 |
| 7296 | TXNRD1 | mRNA | 1.088687494 | 4.35E-154 |
| 1821 | DRP2 | mRNA | 1.7976859 | 0.003195671 |
| 55821 | ALLC | mRNA | -1.029558396 | 0.036328954 |
| 4773 | NFATC2 | mRNA | 1.341338312 | 3.98E-46 |
| 27350 | APOBEC3C | mRNA | 1.072600476 | 0.001674087 |
| 4502 | MT2A | mRNA | 1.422159125 | 4.58E-08 |
| 2118 | ETV4 | mRNA | 2.211770716 | 5.71E-06 |
| 91133 | L3MBTL4 | mRNA | -1.043285995 | 5.03E-13 |
| 3760 | KCNJ3 | mRNA | -1.11214727 | 1.20E-10 |
| 255631 | COL24A1 | mRNA | 4.428362147 | 0.04261996 |
| 8611 | PLPP1 | mRNA | 1.01451947 | 0.015430928 |
| 1728 | NQO1 | mRNA | 2.555102176 | 1.65E-135 |
| 79812 | MMRN2 | mRNA | -1.186500505 | 0.008395129 |
| 438 | ASMT | mRNA | -1.728083479 | 2.16E-04 |
| 23362 | PSD3 | mRNA | -1.144264257 | 1.61E-29 |
| 92092 | ZC3HAV1L | mRNA | -1.267164248 | 0.001070206 |
| 115557 | ARHGEF25 | mRNA | -1.016494847 | 0.031978091 |
| 80726 | IQCN | mRNA | -1.33295385 | 0.008746771 |
