# Supplementary figures and images for: Effects and molecular mechanism of inhibiting p53 signaling pathway by NSUN4 on the resistance to BCL-2 inhibitor for diffuse large B-cell lymphoma
Source: Clin Exp Med. 2026 Mar 22;26(1):204. doi: 10.1007/s10238-026-02117-3 (PMC13038678; doi:10.1007/s10238-026-02117-3)

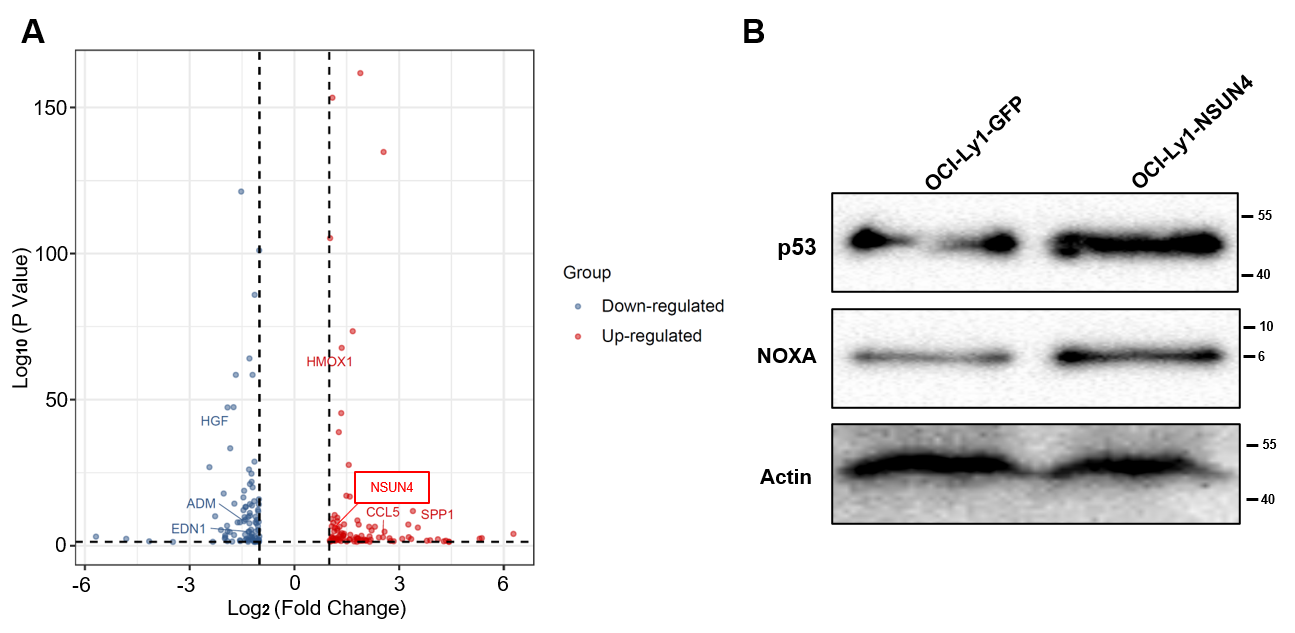

Supplement: Supplementary file 2 — Supplementary Material 2: S Fig. 1 Identification and validation of drug resistance-associated genes. (A) Volcano plot for identifying the most differentially expressed genes. (B) NSUN4 was overexpressing in OCI-Ly1 cells (right). [file 10238_2026_2117_MOESM2_ESM.tif]
